# Reviewer’s dilemma when facing a ‘voluminous’ manuscript

**DOI:** 10.2340/aos.v84.43808

**Published:** 2025-06-10

**Authors:** Shigeki Matsubara

**Affiliations:** a Department of Obstetrics and Gynecology, Jichi Medical University, Tochigi, Japan; b Department of Obstetrics and Gynecology, Koga Red Cross Hospital, Koga, Ibaraki, Japan; c Medical Examination Center, Ibaraki Western Medical Center, Chikusei, Ibaraki, Japan

Dear Editors,

The Editor-in-Chief, Palle Holmstrup [1], highlighted the journal’s difficulty in recruiting qualified reviewers and appealed to researchers’ conscience to accept review invitations. Elin Hadler-Olsen [2] noticed some reasons for declining such requests: the surge of low-quality submissions leads to reviewer fatigue. Hadler-Olsen urged researchers to make greater efforts in crafting manuscripts and called on journals to check article quality more strictly before peer review. I agree with both views. I believe in researchers’ conscience – both as an author and a reviewer. However, relying on it alone may not resolve the current urgency. I offer a tentative proposal: journals should (1) ask authors to shorten overly long or poorly structured manuscripts before review and (2) include word counts and page numbers in reviewer invitations.

Reviewing takes time. Yet, for experienced researchers, it’s usually not difficult when manuscripts are written in an ‘ordinary’ manner [3]. Trouble begins when: (1) the manuscript is overly long and poorly organized, yet (2) it seems to contain something valuable. For instance, a novel concept may be buried in a flood of words, including, for example, excessive ‘research history’.

In reviewing, content takes priority; ‘how it’s written’ comes second. Having reviewed over 2,600 manuscripts (Web of Science record), I prefer not to spend excessive time on unduly long submissions. Often, when I suggest, ‘Revision – make it short’, authors merely cut the ‘history’ sections, leaving the manuscript still lengthy. I then request a ‘re-revision’, finally assess the content – only to find it unworthy. Authors, unaware of the reviewer’s constraints, may feel frustrated by a ‘final rejection’. I understand their feelings, having faced rejection myself while writing over 600 PubMed-indexed articles.

Faced with a lengthy manuscript, many reviewers may think, ‘This will lead to endless revisions. I’d rather not get involved’. They may reject it as ‘not well-written’. But remember: how it’s written is secondary [3]. This approach risks missing the content’s true value, the main focus of evaluation.

Thus, I first suggest that editors ask authors to ‘shorten and organize’ manuscripts before peer review – and, importantly, inform reviewers, ‘This version reflects our request for shortening’. Editors often desk-reject articles that are both (1) poorly organized and (2) low in content. But when only (1) applies and (2) is unclear, reviewers must either invest time in likely endless revisions or reject it, which unfairly burdens them.

Generative AI (GenAI) can shorten manuscripts to any length. This raises two concerns: (1) do authors critically assess GenAI-driven edits and (2) can such revisions truly count as ‘increased readability’, as many journals permit? [4]

Next, reviewer invitations should include word counts and page numbers. Reviewers often decide based on the abstract alone. I recently accepted a request – only to find the manuscript was 100 pages, including supplementary material.

With GenAI driving more submissions, the demand for good reviewers will rise. As Hadler-Olsen stated, obliging reviewers to act like the senior author of the submitted manuscript is the worst scenario. ‘Do not burden reviewers too much’ is the best ‘reward’ at present. This may increase the number of reviewers and improve review quality. I refer to journals in general and do not specifically mean *Acta Odontologica Scandinavica*.

## Conflict of Interest

The author has no conflict of interest.

## Contribution

S.M. identified the significance of this study and wrote the manuscript. In addition, he also ensured that the ICMJE guidelines for authorship are met and agreed to be accountable for all aspects of the work.

## Data availability

Data sharing is not applicable to this article as no new data were created or analyzed in this study.
